# Trophic Niche Overlap in Stingless Bees (*Melipona* Illiger, 1806) Across Seasons in Southern Brazil

**DOI:** 10.1007/s13744-026-01438-y

**Published:** 2026-10-15

**Authors:** Amanda Garçoa Raulik, Luiz Gabriel Ludwig, Adriano Silvério, João M. D. Miranda

**Affiliations:** 1 Post Graduate Program in Zoology, Universidade Federal Do Paraná, Avenida Cel. Francisco H. Dos Santos 100, Curitiba, PR 81530-000 Brazil; 2 Department of Biological Sciences, Universidade Estadual Do Centro-Oeste, Alameda Élio Antônio Dalla Vecchia 838, Guarapuava, PR 85040-167 Brazil

**Keywords:** Meliponiculture, Niche overlap, Pollination, Seasonality, Trophic ecology

## Abstract

Understanding how stingless bee species share floral resources is essential for optimizing meliponiculture and supporting pollinator conservation. This study investigated the trophic niche overlap of four *Melipona* species (*M. quadrifasciata*, *M. bicolor*, *M. torrida*, and *M. mondury*) in Southern Brazil across warm and cold seasons. Pollen samples were collected monthly over 10 months and analyzed to assess richness, trophic niche breadth, and overlap between species’ trophic niches. Results showed seasonal variation in foraging patterns, with all species (except *M. mondury*) reducing their niche breadth in colder months. *Melipona mondury*, a species native to warmer regions of the Atlantic Forest and lacking reproductive diapause, maintained a broad niche year-round, possibly increasing its vulnerability in colder climates. The highest overlap occurred between *M. quadrifasciata* and *M. mondury* during the cold season, raising concerns about managing these species in consorted meliponiculture systems. The most frequently visited botanical families included Myrtaceae, Arecaceae, Asteraceae, Moraceae, and Fabaceae highlighting their importance as key floral resources. These findings highlight the importance of considering seasonal dynamics and species-specific traits in meliponiculture, including the need for targeted supplemental feeding and resource planning. Promoting key botanical resources such as Myrtaceae and Arecaceae can support sustainable colony development in high-density environments.

## Introduction

The Neotropical region hosts the richest bee fauna in the world, and bees play a vital role in maintaining plant biodiversity (Moure et al. 2007). Among them, stingless bees of the tribe Meliponini stand out for their high diversity, abundance, and major contribution to plant pollination in tropical and subtropical ecosystems (Nates-Parra et al. 2008). Within this group, bees of the genus *Melipona* are among the most widely recognized and managed species, both because of their ecological importance and their value in meliponiculture (Jaffé et al. 2015).

Meliponiculture, the rational management of stingless bees, has expanded in recent decades as an activity of economic, cultural, and conservation interest (Nogueira-Neto et al. 1986). Several *Melipona* species are managed for the production of honey, pollen, wax, and propolis, and their maintenance in meliponaries has also been regarded as a strategy to support the conservation of native bees (Reyes-González et al. 2016; Gemim & de Melo Silva 2017). However, unlike natural conditions, meliponaries may concentrate multiple colonies, often belonging to different species, within the same area. This artificial increase in colony density may modify how floral resources are shared among bees, especially when foragers from different species exploit the same surrounding vegetation.

Trophic niche breadth and trophic niche overlap are central concepts for understanding how species partition and share resources within a community (Colwell & Futuyma 1971). Niche breadth reflects how restricted or flexible a species is in its use of available resources, and may vary in response to resource availability, competition, and seasonal changes in the plant community (Heithaus 1979). Niche overlap, in turn, indicates the extent to which species rely on the same resources, and high overlap may signal potential competition, particularly when resource availability is reduced. Importantly, these parameters are not static: seasonal variation in flowering phenology can shift both the diversity of resource-use strategies and the degree of dietary specialization, leading to temporal fluctuations in overlap and breadth among co-occurring species (Blüthgen & Klein 2011). Together, these metrics help reveal whether coexisting species tend to partition floral resources or exploit them redundantly, and how such patterns change as resource availability varies over time.

These ecological processes are highly relevant to colony management in meliponaries. When multiple species are kept in the same area, high niche overlap may reflect stronger competition for key floral resources, especially during periods of reduced availability. This situation can impair colony performance and increase the risk of pollination deficits, with potential ecological and economic consequences (Biesmeijer and Slaa 2006). In contrast, broader niche breadth may indicate greater flexibility in resource use, which can help buffer colonies against seasonal shortages. From this perspective, understanding patterns of resource use can support more informed management decisions, including which species can be maintained together, how many colonies a given area can support, and whether the surrounding vegetation provides sufficient and diverse floral resources throughout the year. Thus, knowledge of floral resource use is important not only for understanding the trophic ecology of managed bees, but also for supporting conservation-oriented meliponiculture (Aguiar et al. 2013).

Despite the growing importance of meliponiculture, there is still limited information on how commonly managed *Melipona* species share trophic resources across seasons when occurring in the same managed environment. This is particularly relevant for species that are frequently kept together in meliponaries but may differ in morphological traits, such as body size and coloration, and in their potential use of floral resources.

In this study, we evaluated seasonal trophic niche overlap among four stingless bee species of the genus *Melipona* (*Melipona quadrifasciata, M. bicolor, M. torrida, and M. mondury*), which are commonly kept together in meliponaries. Specifically, we asked: (1) how do the floral resource choices of these four species vary between warm and cold seasons, and how does seasonality influence trophic niche breadth and overlap among them? and (2) do body size, coloration, and phylogenetic distance influence trophic niche overlap among these species? By linking seasonal resource use and species traits to the dynamics of floral resource sharing, we aimed to contribute both to the trophic ecology of stingless bees and to the ecological basis of meliponary management.

## Methods

### Study area

**Fig. 1:**
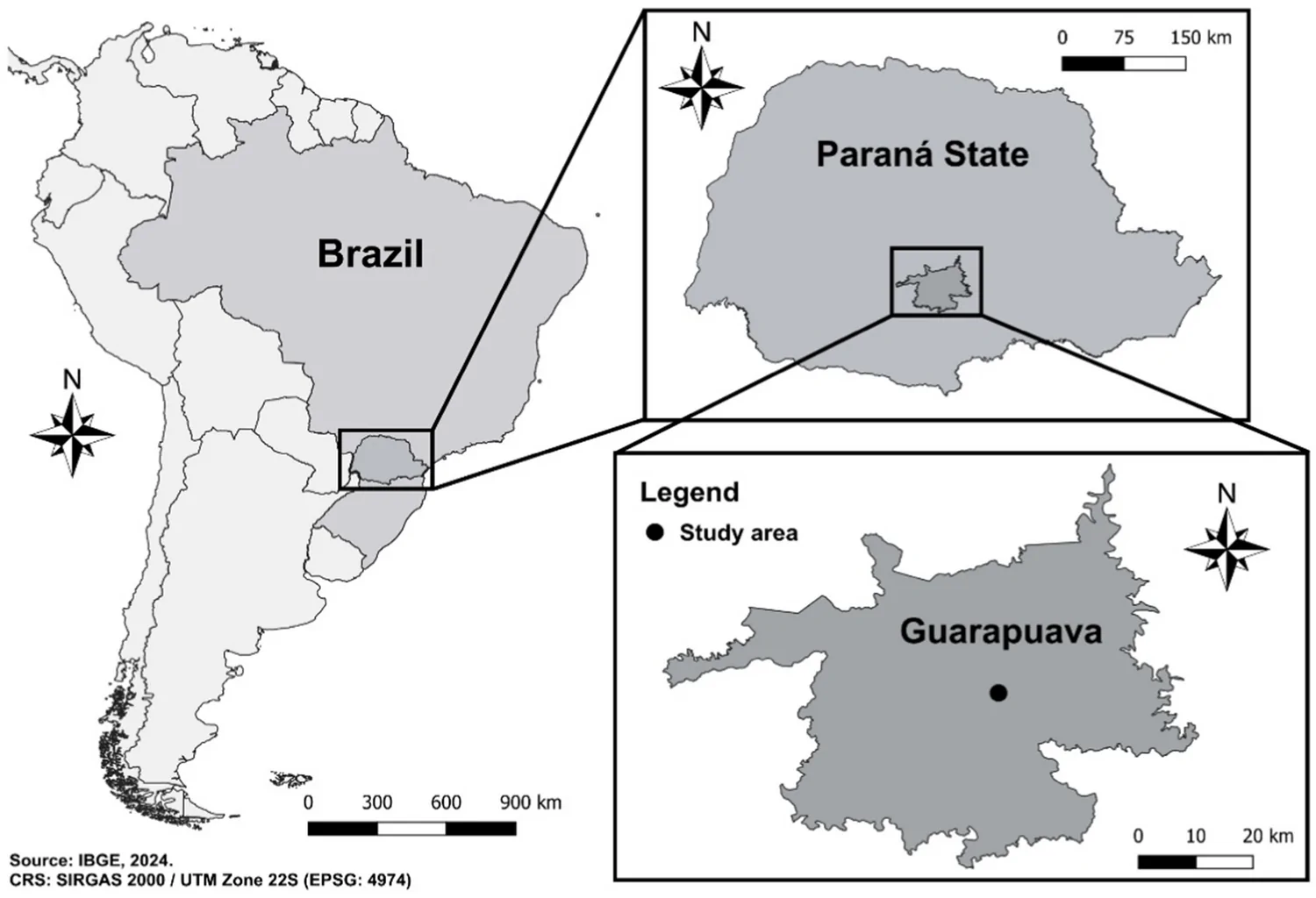
Location of the study area in the municipality of Guarapuava, state of Paraná, southern Brazil, where the data were collected. The marked point represents the sample collection site. Base map: IBGE (2024). Reference system: SIRGAS 2000/UTM Zone 22S (EPSG: 4974)

The study was carried out at the Meliponário Abelhas da Mata, located in the municipality of Guarapuava, Paraná, Brazil (25°22′23,69″S; 51°26′17,967″W, 1.119 m altitude), in a peri-urban region of the city (Fig. 1). The region is located in a transitional area between Araucaria Moist Forest (Mixed Ombrophilous Forest) and high-altitude grasslands, originally characterized by natural fields interspersed with patches of Atlantic Forest. The climate is classified as humid subtropical and mesothermal, with an average annual temperature of 16.8 °C (Costa and Andrade 2017), corresponding to the Cfb type in the Köppen–Geiger climate classification. Rainfall seasonality in southern Brazil is regionally heterogeneous. In Guarapuava, located in the central-southern highlands of Paraná, precipitation occurs throughout the year, although rainfall is typically higher in spring and summer and a sharply defined dry season is not observed (Pereira et al. 2008).

### Stingless bee species selected

The four species chosen for this study are endemic to the Atlantic Forest (Nieh et al. 2003; Camargo et al. 2013; Lopes et al. 2022), commonly bred together in meliponaries (Nogueira-Neto 1997) and were housed in boxes of wood for over a year at the meliponary. These target species were *Melipona quadrifasciata quadrifasciata* Lepeletier, 1836; *Melipona bicolor schencki* Gribodo, 1896; *Melipona torrida* Friese, 1916; and *Melipona mondury* Smith, 1893. The selection of these stingless bee species was based on two criteria: (1) their common use in consorted meliponiculture and (2) the differences in foraging activities, reflected in their flight ranges and the resource selection. Although the four species are considered generalists, we evaluated three aspects such as size and color of the body and phylogenetic distance between them.

### Stingless bee monitoring and sample processing

In the study region, located in the central-southern highlands of Paraná, a sharply defined dry season is not observed, although rainfall tends to increase during spring and summer (Pereira et al. 2008). Therefore, sampling was not divided into dry and rainy seasons, but into warm (spring–summer) and cold (autumn–winter) periods. Field sampling was conducted monthly from November 2022 to August 2023, providing coverage of both seasonal periods considered in the study. Although September and October were not included due to logistical constraints, the sampling design still encompassed representative months of both warm and cold seasons. Each sampling event consisted of standardized collections performed under suitable weather conditions (temperatures between 18 and 20 °C and absence of rainfall), when stingless bees are known to be most active (Hilário et al. 2000).

For each of the four focal species, pollen was collected from five foraging individuals originating from five distinct colonies, totaling 20 samples per month. Sampling effort was evenly distributed across the study period, resulting in 200 pollen samples in total. While most collections could be completed within a single field day, during colder months additional sampling time was occasionally required to ensure sufficient representation of all species. To obtain the pollen samples used by stingless bees, the entrances were blocked with small sticks, preventing forage bees from entering. As soon as the foraging bees approached and tried to enter the hive, only those that were carrying pollen on their corbiculae were selected and captured. This capture was done manually or with the help of a small plastic container with a lid. The collected pollen was stored in microtubes (1 ml), containing AFA solution (5% acetic acid, 5% formaldehyde, and 90% of 50% alcohol), and the bees were released to return to their activities.

### Palynological analysis

The samples underwent the acetolysis process described by Erdtman (1952) with minor modifications. Semi-permanent slides were prepared using glycerinated gelatin and examined under bright-field microscopy. Pollen grains were identified to the lowest possible taxonomic resolution, predominantly at the family level (Appendix). Taxonomic classification follows the Angiosperm Phylogeny Group (2016).

The pollen identification was conducted with the support of a botanical specialist and virtual platforms such as Online Pollen Catalog Network (RCPol) and Global Pollen Project (Martin & Harvey 2017). Diagnostic features considered included pollen size, shape, aperture type and configuration, and exine ornamentation.

### Morphological characterization of stingless bees

Ten individuals from each of the four stingless bee species were collected at the entrance of their nests. Each specimen was euthanized in a killing chamber using ethyl acetate and was measured for its mass (mg) using a precision scale. The average weight of the 10 individuals from each species was used as a proxy for body size. Regarding coloration, one representative specimen per species was analyzed and classified based on the percentage of the body surface (dorsal view) displaying light and dark colors, using the R packages *magick* v. 2.8.5 (Ooms 2021) and *imager* v.1.0.2 (Barthelme and Tschumperlé 2019). This analysis was intended to provide a representative overview of interspecific differences in coloration patterns.

### Phylogenetic distance

To calculate the evolutionary or phylogenetic distance among the four stingless bee species, the molecular phylogeny of *Melipona* proposed by Ramírez et al. (2010) was used. The phylogeny was reconstructed as an ultrametric tree using the *chronos* function from *ape* v. 5.0 package in R (Paradis & Schliep 2019), and phylogenetic distances between species pairs were calculated based on branch lengths. To update the nomenclature and evolutionary relationships, substitute taxa were used for species absent from the original phylogeny: *Melipona quadrifasciata anthidioides* was used as a substitute for *M. quadrifasciata quadrifasciata*, given the close evolutionary relationship between subspecies of *M. quadrifasciata* (Batalha-Filho et al. 2010); *M. marginata* was used as a substitute for *M. torrida* since the latter replaced the name previously given to *M. marginata obscurior*, a former subspecies of *M. marginata* (Melo 2013); and *M. rufiventris rufiventris* was used as a substitute for *M. mondury* since *M. mondury* was previously considered a subspecies of *M. rufiventris* (Melo 2003). In each case, the substitute taxon was selected as the closest available representative in the phylogeny, based on taxonomic and nomenclatural evidence of evolutionary proximity.

### Data analysis

For each stingless bee species, both pollen richness (i.e., the number of pollen morphotypes) and feeding niche breadth were calculated. Niche breadth was calculated using Levins’ index (B), with the standardized value indicated as BA.

Based on the relative abundances of pollen morphotypes, trophic niche overlap among the four stingless bee species was analyzed. Niche overlap for warm season and cold season was assessed using Pianka’s Index and a Non-Metric Multidimensional Scaling (NMDS) perceptual map. A permutation test with 1000 randomizations was applied to evaluate whether the observed niche overlaps were significantly higher than expected by chance. All analyses were conducted in R using the *vegan* v. 2.5–5 package (Oksanen et al. 2019).

Based on the size and color data of the four stingless bee species (Table 1), two distance matrices were constructed: one for size distance and one for color distance, both based on Euclidean Distance. Additionally, from the phylogeny of the genus *Melipona* (Ramírez et al. 2010), a phylogenetic distance matrix was constructed for the four stingless bee species.

**Table 1:** Morphological character matrix of the four analyzed stingless bee species (*Melipona quadrifasciata*, *Melipona bicolor*, *Melipona torrida*, and *Melipona mondury*)

Bee species	Average weight (mg)	Dark coloration (%)	Light coloration (%)
*M. quadrifasciata*	62.1 ± 4.04	87.21	12.79
*M. bicolor*	51.1 ± 4.43	90.21	9.78
*M. torrida*	27.2 ± 2.44	86.51	13.49
*M. mondury*	60.6 ± 8.49	2.55	97.45

To test which variables best explain dietary niche overlap among the four stingless bee species, we employed a multiple regression on distance matrices (MRM) approach. The predictor variables were size distance (DSize), color distance (DColor), and phylogenetic distance (DPhyl). This analysis was performed using the *ecodist* v. 2.1.3 package in R (Goslee & Urban 2007).

## Results

**Fig. 2:**
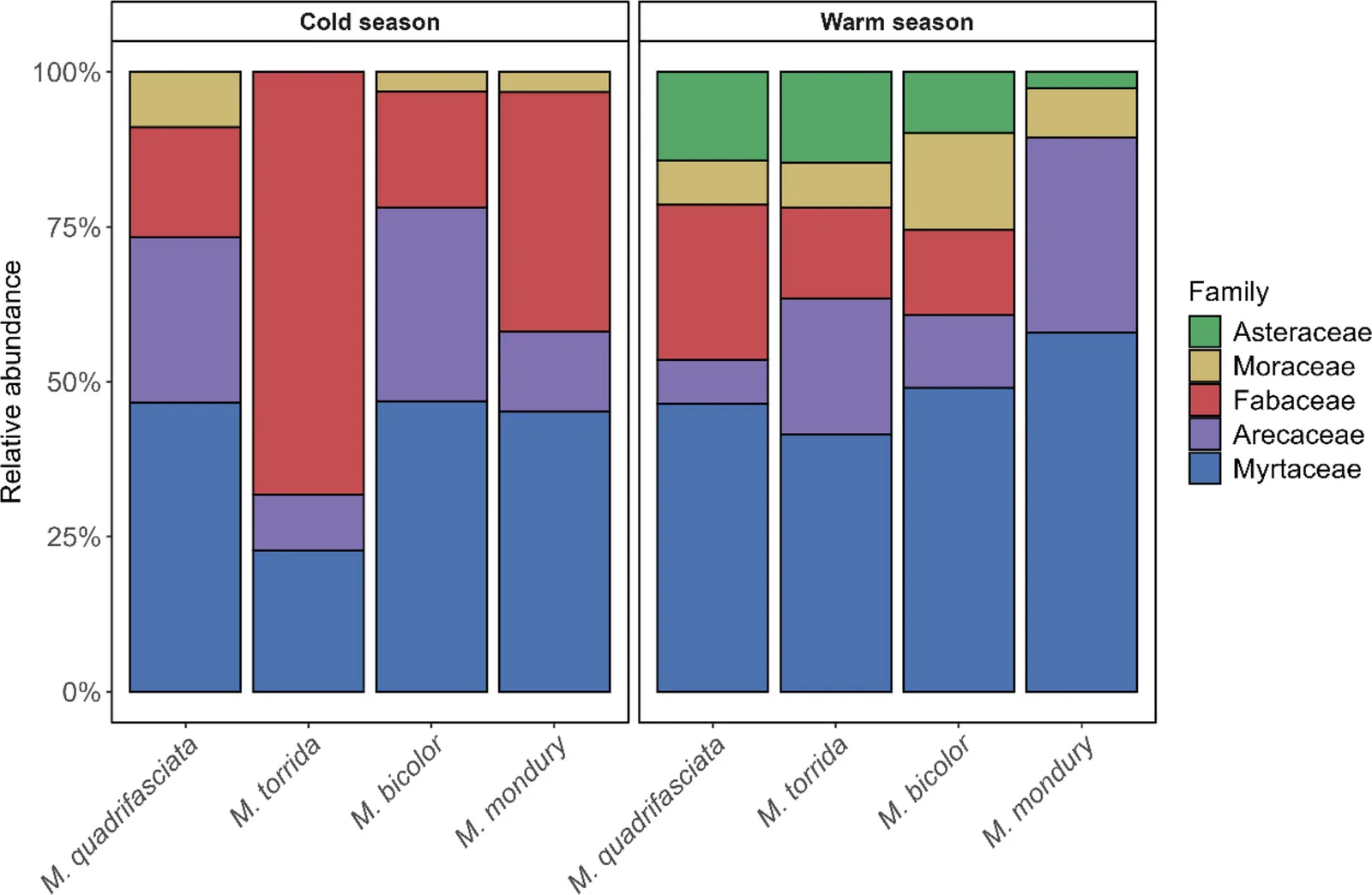
Relative abundance of pollen from the five most representative plant families found in the diet of four stingless bee species (*Melipona quadrifasciata*, *Melipona torrida*, *Melipona bicolor*, and *Melipona mondury*) during the cold and warm seasons

During the warm season, *Melipona bicolor* exhibited the highest richness of pollen morphotypes used (S = 17), followed by *M. torrida* (S = 15), while *M. mondury* showed the lowest richness (S = 9). In terms of trophic niche breadth, *M. quadrifasciata* presented the highest value (B = 10.1), whereas *M. mondury* had the lowest (B = 3.5) (Table 3). In the cold season, richness values were lower for some species, with *M. mondury* maintaining the same richness recorded in the warm season (S = 9), *while M. torrida* used 8 pollen morphotypes and *M. bicolor* showed the lowest richness in this season (S = 6) (Table 2). The most represented plant family in the pollen samples was Myrtaceae, which dominated the diets of all species. Other frequent families included Arecaceae, Fabaceae (including morphotypes assigned to Mimosoideae), Moraceae, and Asteraceae. Asteraceae was recorded only during the warm season and occurred in the diets of all species, although its relative contribution was higher in *M. quadrifasciata* and *M. torrida* (Fig. 2).

**Table 2:** Trophic niche breadth values (Levins’ index: B and standardized BA), richness of visited plant morphotypes (S), and pairwise niche overlap values (Pianka’s index) among stingless bee species reared in meliponaries in southern Brazil during the warm season. *p*-values are shown only for statistically significant pairwise niche overlap comparisons, based on permutation tests with 1000 randomizations

	**B**	**BA**	**S**	**Niche overlap (warm season)**			
				*M. quadrifasciata*	*M. torrida*	*M. bicolor*	*M. mondury*
*M. quadrifasciata*	10.1	0.36	16	-	**0.77 (*p* = 0.004)**	**0.90 (*p* = 0.001)**	**0.70 (*p* = 0.006)**
*M. torrida*	9.2	0.32	15	0.77	-	**0.85 (*p* = 0.003)**	**0.86 (*p* = 0.001)**
*M. bicolor*	8.3	0.29	17	0.90	0.85	-	**0.82 (*p* = 0.001)**
*M. mondury*	3.5	0.10	9	0.70	0.86	0.82	-

### Seasonal variation

**Fig. 3:**
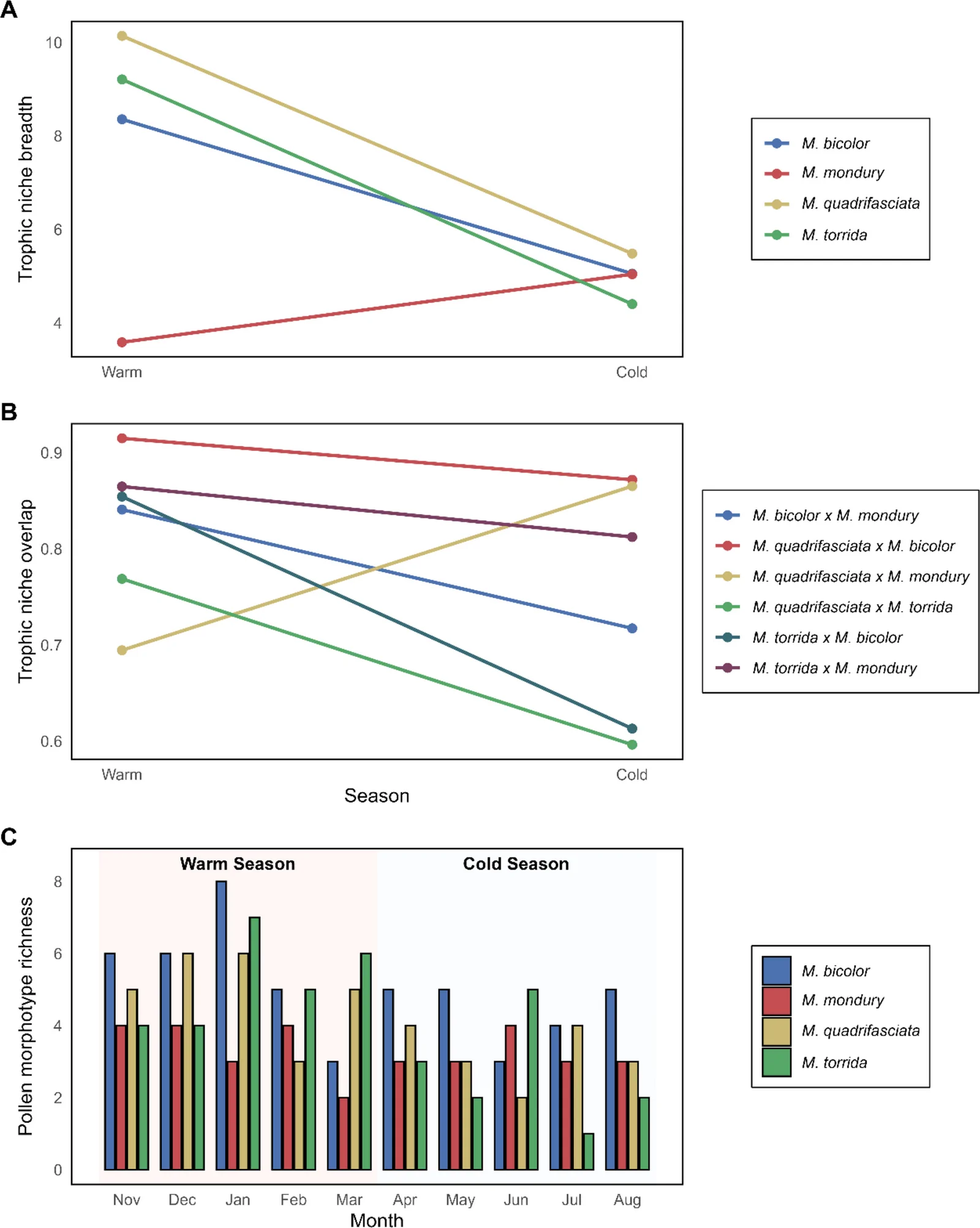
**A** Trophic niche breadth (Levins’ index),** B** trophic niche overlap (Pianka’s index) between pairs of stingless bee species in warm and cold season and** C** richness of pollen morphotypes per month throughout the study

Throughout the year, *M. mondury* collected significantly fewer pollen morphotypes than *M. quadrifasciata, M. bicolor*, and *M. torrida*. Similarly, *M. mondury* also exhibited lower trophic niche breadth compared to the other species (Fig. 3A). This same pattern was observed during the warm season. In contrast, during the cold season, the values of pollen morphotype richness and trophic niche breadth were more similar among species (Tables 2 and 3). In both seasons, the highest trophic niche overlap was observed between *M. quadrifasciat*a and *M. bicolor* (91% in the warm season and 87% in the cold season), indicating resource sharing between these species. During the warm season, all species pairs exhibited significantly higher niche overlap than expected by chance (*p* < 0.01 for all pairs), with particularly high values between *M. torrida* and *M. mondury* (86%, p = 0.001), and between *M. bicolor* and *M. torrida* (85%, *p* = 0.003). The lowest overlap occurred between *M. quadrifasciata* and *M. mondury* (69%, *p* = 0.006).

**Table 3:** Trophic niche breadth values (Levins’ index: B and standardized BA), richness of visited plant morphotypes (S), and pairwise niche overlap values (Pianka’s index) among stingless bee species reared in meliponaries in southern Brazil during the cold season. *p*-values are shown only for statistically significant pairwise niche overlap comparisons, based on permutation tests with 1000 randomizations

	**B**	**BA**	**S**	**Niche overlap (cold season)**			
				*M. quadrifasciata*	*M. torrida*	*M. bicolor*	*M. mondury*
*M. quadrifasciata*	5.4	0.49	8	-	0.59	**0.87 (*p* = 0.017)**	**0.86 (*p* = 0.026)**
*M. torrida*	4.4	0.37	8	0.59	-	0.61	0.81
*M. bicolor*	5	0.45	6	0.87	0.61	-	0.71
*M. mondury*	5	0.44	9	0.86	0.81	0.71	-

**Fig. 4:**
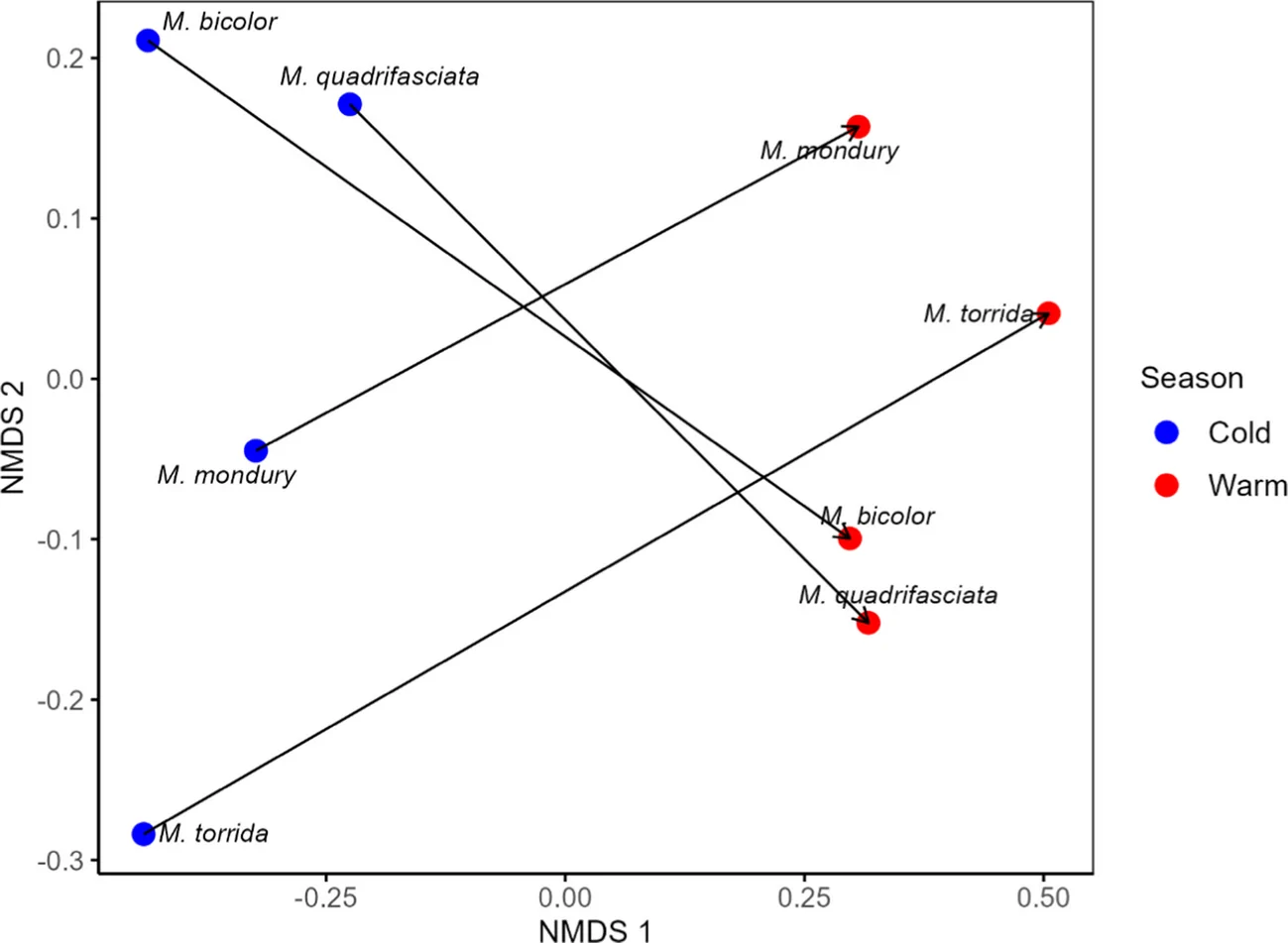
Trophic niche overlap of the four stingless bee species in the warm season (red label) and the cold season (blue label), based on non-metric multidimensional scaling (NMDS) analysis using the Pianka’s similarity index

In contrast, during the cold season, only two species pairs exhibited significant niche overlap: *M. quadrifasciata* with *M. bicolor* (87%, *p* = 0.017) and with *M. mondury* (87%, *p* = 0.026). Other pairs, such as *M. quadrifasciata* and *M. torrida* (60%, *p* = 0.27), and *M. torrida* and *M. bicolor* (61%, *p* = 0.22), did not show significant overlap, suggesting more distinct foraging patterns in those interactions during this period (Fig. 4). Overall, in the cold season, most stingless bee species showed lower values of pollen morphotype richness and trophic niche breadth than in the warm season. However, *M. mondury* did not follow this same trend (Fig. 3C).

The multiple regression on distance matrices (MRM) analysis did not reveal any significant relationships between the predictor variables and trophic niche overlap among the stingless bee species (Table 4). Size distance, color distance, and phylogenetic distance were not significantly associated with variations in trophic niche overlap when considering all samples combined, nor in the separate analyses for the warm and cold seasons.

**Table 4:** Coefficients of the predictor variables for the multiple regression on distance matrices (MRM) predicting trophic niche overlap in four stingless bee species from the Brazilian Atlantic Forest (*Melipona quadrifasciata*, *Melipona bicolor*, *Melipona torrida*, and *Melipona mondury*). DPhyl = Phylogenetic distance; DSize = Size distance; DColor = Color distance

	**Coefficient**	***p*-value**
Intercept	0.9247081	0.3713
DColor	− 0.0002268	0.7247
DSize	− 0.0019988	0.1071
DPhyl	0.0163249	0.6726

## Discussion

Climatic factors are intrinsically linked to the availability and density of resources for stingless bees (Keppner and Jarau 2016; Moura et al. 2022). Most studies evaluating the effects of seasonality on niche overlap in stingless bees analyze similarities and differences between the rainy and dry seasons (Brito et al. 2012; Santos et al. 2013; Escobedo-Kenefic et al. 2020; Ferreira et al. 2023). The results presented here demonstrate that stingless bees follow the pattern observed in previous studies, foraging more intensively for pollen resources during the warm season, due to increased floral abundance and higher temperatures (Souza et al. 2006; Ferreira-Junior et al. 2010). In contrast, during the cold season, a reduction in species richness and trophic niche breadth was observed in three of the studied species—Melipona* quadrifasciata, M. bicolor*, and *M. torrida—all* of which conformed to this pattern. However, *M. mondury* deviated from it, maintaining similar niche breadth values across both seasons, suggesting a distinct foraging strategy. This behavior may be related to its biogeographic origin: although native to the Atlantic Forest, *M. mondury* is not originally found in the study region, being more commonly distributed in lower-altitude areas of the Atlantic Forest along the Brazilian coast, where the climate is warmer and more humid (Mouga 2009; Mouga and Dec 2015; da Silva Mouga 2015). Furthermore, this species does not undergo reproductive diapause, which sustains its year-round demand for pollen and may increase its vulnerability in subtropical climates with pronounced winters. The increase in niche overlap between *M. quadrifasciata* and *M. mondury* during cold season may lead to challenges in colony development due to the reduced availability of floral resources during this period. These findings have important implications for meliponiculture, particularly regarding colony maintenance during colder periods. Supplemental feeding, including sugar syrup and pollen-based food, may be essential to support the survival and proper development of colonies maintained outside their natural range.

Some botanical taxa form the basis of stingless bees’ diet (e.g., Myrtaceae, Arecaceae), while others are used more occasionally, which helps explain the trophic relationships observed in the networks of both warm and cold seasons. During the cold season, the availability of flowering plants decreases, which strengthens the bee–plant interactions (Ferreira et al. 2023). Additionally, several taxa recorded correspond to herbaceous species likely native to grasslands or forest edges (i.e., Asteraceae, Begoniaceae, Lamiaceae, Urticaceae, Verbenaceae) reflecting the transitional nature of the study area between Araucaria Moist Forest (Mixed Ombrophilous Forest) and high-altitude grasslands.

The concept of a key resource can be understood as a food resource capable of determining the survival potential of organisms. Additionally, it can influence the composition of biological communities, as its absence or low availability may affect species’ population density, long-term viability, or even lead to local extinction (Primack 2014). Stingless bees exhibit a certain affinity for resources considered key to colony maintenance. The collection of morphotypes from botanical families that flower abundantly throughout the year, such as Myrtaceae, facilitates species coexistence in environments like meliponaries, where there is an artificially increased local density of bees. Identifying key resources may therefore serve as an important tool for effective long-term conservation and management strategies.

Bees of the genus *Melipona* exhibit social behavior and live in populous, perennial colonies, requiring them to exploit a wide range of floral resources throughout the year. Most flowering plants are ephemeral sources, blooming for only a few days (Ramalho et al. 2007). Therefore, a generalist foraging strategy is considered the norm among eusocial bees of the family Apidae. Their foraging patterns vary seasonally in response to environmental factors such as flowering periods—with summer and spring being seasons of abundant floral availability (Winston 2003). *Melipona* bees have been recorded visiting a variety of botanical families, depending on the surrounding vegetation (Bueno et al. 2023). Although stingless bees forage on many species from different plant families, some families are more frequently utilized. Floristic surveys conducted in the Araucaria Moist Forest have identified the Myrtaceae family as one of the richest or most abundant (Cordeiro and Rodrigues 2007; Albuquerque et al. 2011; Vieira and May 2020) with about 246 species belonging to 20 genera recorded solely within the Atlantic Forest domain, and 220 species belonging to 16 genera recorded in Paraná state alone (Flora do Brasil 2026). In the Atlantic Forest, Meliponini bees frequently visit flowers of *Myrcia tomentosa*, particularly species of *Melipona* and *Plebeia* (Ramalho 2004). In São Paulo, *Melipona mondury* (previously cited as *M. rufiventris*) has been identified as one of the most effective pollinators of Myrtaceae species (Fidalgo 2002). Other studies have consistently reported Myrtaceae as one of the most visited plant families by bees in Brazil (Carvalho et al. 2001; Antonini et al. 2006; Ramalho et al. 2007), with *Melipona* species being the most frequent pollinators of Myrtaceae in the country. This is largely attributed to the presence of nectaries in these plants, which offer nectar in addition to pollen (Antonini et al. 2006; Gressler et al. 2006).

The Arecaceae family exhibits asynchronous flowering, providing floral resources during several months of the year. In Brazil, approximately 270 species and 38 native genera are recognized, standing out for their abundance and species richness in Neotropical forests (Lara et al. 2017). In the state of Paraná, 35 species distributed among 19 genera have been reported (Flora do Brasil 2026). The importance of Arecaceae in the pollen diet of *Melipona* bees has already been highlighted by Oliveira et al. (2009). The Araucariaceae family, in turn, was recorded exclusively in the samples of *M. mondury* and is noteworthy since these plants do not produce flowers, yet their pollen is protein-rich and nutritionally valuable. Other studies have also reported the use of anemophilous plants (e.g., Cecropiaceae) by stingless bees in the Atlantic Forest (Morgado et al. 2011; Braga et al. 2014).

The trophic niche breadth of the four stingless bee species endemic to the Atlantic Forest analyzed in this study reveals a generalist feeding behavior, which is common among eusocial bees and reflects their plasticity in the use of pollen resources. Body size and coloration are important traits in the trophic niche segregation of stingless bees (Hilário et al. 2001; Pereboom and Biesmeijer 2003; Teixeira and Campos 2005; Biesmeijer and Slaa 2006). However, these traits were not effective in predicting the niche overlap observed in this study. This may be due to the fact that the analysis included only four species from the same genus, *Melipona*. Although the *Melipona* genus is highly diverse, the selected species are among the largest stingless bees and show little variation in body size, as well as a close evolutionary relationship. Despite morphological differences, this variation could not be linked to the patterns of niche overlap among the analyzed species. Expanding the sampling to include species from other genera, with greater variation in body size, coloration, and phylogenetic distance, may help clarify these dietary differences more effectively. Additionally, September and October were not sampled due to logistical constraints, which may have limited the representation of floral resource availability during the transition to the warm season.

The results obtained in this study can directly support the management and strategic organization of meliponaries located in the Atlantic Forest ecosystem, particularly in colder regions such as the Araucaria Moist Forest. The identification of key resources, such as plant species from the Myrtaceae and Arecaceae families, which proved highly attractive to the studied bee species, suggests that cultivating these plants may serve as an important nutritional support strategy throughout the year. Moreover, data on niche overlap indicate that the coexistence of certain species may require special attention. For example, *M. quadrifasciata* and *M. mondury* showed a marked increase in niche overlap during the cold season, suggesting that keeping both species in the same meliponary should be approached with caution. If this combination is adopted, it is recommended to increase the availability of supplemental feeding for both species. It is also noteworthy that *M. mondury* exhibited a distinct foraging strategy, maintaining a wide niche breadth even in the cold season, likely due to the absence of reproductive diapause. This trait may render the species more vulnerable in colder climates, as it must maintain a high demand for pollen even when floral resources are scarce, which could compromise colony viability without adequate support.

## Data Availability

The data that support the findings of this study are available from the corresponding author upon reasonable request.

## Appendix

Table 5.

**Table 5:** Occurrence of pollen types identified in the samples collected from November 2022 to August 2023 (warm and cold seasons)

Botanical taxa	*M. quadrifasciata*		*M. torrida*		*M. bicolor*		*M. mondury*	
	Warm	Cold	Warm	Cold	Warm	Cold	Warm	Cold
Amaryllidaceae	0	0	2	0	0	0	0	0
Araucariaceae	0	0	0	0	0	0	1	0
Arecaceae	2	12	9	2	6	10	12	4
Asteraceae–*Ambrosia* spp.	0	0	2	0	2	0	0	0
Asteraceae–*Baccharis* spp.	3	0	4	0	3	0	1	0
Asteraceae–*Dahlia* spp.	1	0	0	0	0	0	0	0
Begoniaceae	1	0	1	0	1	0	0	2
Betulaceae	0	3	0	0	1	0	0	0
Cardiopteridaceae	0	0	1	0	0	0	0	0
Cyperaceae	0	0	1	0	0	0	0	0
Euphorbiaceae	4	0	2	1	5	1	2	1
Fabaceae–Mimosoideae–*Piptadenia* spp.	1	0	0	0	1	0	0	0
Fabaceae–Mimososideae–*Mimosa* spp.	3	7	0	10	2	1	0	10
Fabaceae	3	1	6	5	4	5	0	2
Griselinaceae	1	0	0	0	0	0	0	0
Lamiaceae	0	0	1	0	0	0	0	0
Moraceae	2	4	3	0	8	1	3	1
Myrtaceae 1	5	0	2	0	8	0	2	0
Myrtaceae 2	7	13	11	5	15	11	18	10
Myrtaceae 3	1	8	4	0	2	4	2	4
Rosaceae	2	0	1	0	2	0	0	0
Rutaceae	0	0	2	0	0	0	0	0
Solanaceae	0	2	1	6	0	6	0	1
Urticaceae	0	0	0	0	1	0	0	0
Valerianaceae	0	0	0	0	1	0	1	0
Verbenaceae	1	0	0	0	0	0	0	0
